# Supplementary material for: Nanodrugs for the Treatment of Ischemic Stroke: A Systematic Review
Source: Int J Mol Sci. 2023 Jun 28;24(13):10802. doi: 10.3390/ijms241310802 (PMC10341504; doi:10.3390/ijms241310802)
Supplement: Supplementary file 1 [file ijms-24-10802-s001.zip › Supplementary Table S1.pdf]

Table S1. The search criteria.

Pub Med	
Search strategies	Search keywords
1.Nano-drug	(nanoparticle) OR (nanomaterial) OR (nanodrugs)
2.Nano-Treatment	(nanotreatment) OR(nanomedicine) OR (nanotechnology) OR (nanobiotechnology)
3. Ischemic stroke	(Stroke) OR (ischemic stroke) OR (Brain Ischemia) OR (cva) OR (ischemic strokes) OR (stroke syndrome) OR (Brain Ischemia) OR (cerebral vascular accident) OR (cerebrovascular accident) OR (cerebrovascular accidents) OR (cerebral vascular accidents) OR (Brain Ischemias) OR (cerebral ischemia) OR (cerebral ischemias) OR (ischemic brain) OR (ischemic brains)
Web Of Science	
Search strategies	Search keywords
1.Nano-drug	(nanoparticle) OR (nanomaterial) OR (nanodrugs)
2.Nano-Treatment	(nanotreatment) OR (nanomedicine) OR (nanotechnology) OR (nanobiotechnology)
3. Ischemic stroke	(Stroke) OR (ischemic stroke) OR (Brain Ischemia) OR (cva) OR (ischemic strokes) OR (stroke syndrome) OR (Brain Ischemia) OR (cerebral vascular accident) OR (cerebrovascular accident) OR (cerebrovascular accidents) OR (cerebral vascular accidents) OR (Brain Ischemias) OR (cerebral ischemia) OR (cerebral ischemias) OR (ischemic brain) OR (ischemic brains)
