# Supplementary material for: Nanodrugs for the Treatment of Ischemic Stroke: A Systematic Review
Source: Int J Mol Sci. 2023 Jun 28;24(13):10802. doi: 10.3390/ijms241310802 (PMC10341504; doi:10.3390/ijms241310802)
Supplement: Supplementary file 1 [file ijms-24-10802-s001.zip › Supplementary Table S2.pdf]

Table S2. Inclusion and exclusion criteria.

Inclusion Criteria	Exclusion criteria
1) Studies that used in vivo models of ischemic brain lesions*	1) In vitro studies that used any microorganism or any animal or human-derived cell lines
2) Studies that used nanoparticles loaded or not with some type of drug for stroke treatment	2) Studies that used in vivo models with other types of brain injuries than ischemic lesions (e.g., hemorrhagic stroke, brain tumor, neurodegenerative diseases)
*In vivo models represent all experimental animal types, regardless of species, sex, age, or stage of life. Studies that include in vivo treatment of animals but evaluate some or all of the effects on derived cells ex vivo will also be considered in vivo studies.	3) Studies that used nanotechnology for paraclinic exploration
	4) Studies that used stroke drug therapy on a larger scale than the nanoscale
