# Supplementary material for: Nanodrugs for the Treatment of Ischemic Stroke: A Systematic Review
Source: Int J Mol Sci. 2023 Jun 28;24(13):10802. doi: 10.3390/ijms241310802 (PMC10341504; doi:10.3390/ijms241310802)
Supplement: Supplementary file 1 [file ijms-24-10802-s001.zip › Supplementary Table S3.pdf]

Table S3. Quality Assessment of Each Study Included in the Systematic Review by Using the the SYRCLE criteria.

Article	Item of the SYRCLE criteria									
	1	2	3	4	5	6	7	8	9	10
1	YES	NK	NO	NK	NK	NK	NK	NO	YES	NK
2	YES	YES	NK	YES	NK	YES	NK	NO	YES	NK
3	YES	YES	YES	YES	YES	YES	YES	YES	YES	NK
4	YES	YES	YES	YES	YES	YES	YES	YES	YES	NK
5	YES	YES	YES	YES	YES	YES	YES	YES	YES	NK
6	YES	YES	YES	YES	NK	YES	NK	NO	YES	NK
7	YES	NO	YES	YES	YES	YES	YES	YES	YES	NK
8	YES	YES	NK	NK	NK	NK	NK	NO	NK	NK
9	YES	YES	YES	YES	YES	YES	YES	YES	YES	NK
10	YES	YES	NK	NK	NK	NK	NK	YES	NK	NK
11	YES	YES	NK	YES	YES	YES	YES	YES	YES	NK
12	YES	YES	NK	NK	YES	NK	YES	NK	YES	NK
13	YES	YES	NK	NK	YES	NK	YES	YES	YES	NK
14	YES	YES	NK	YES	NK	YES	NK	YES	YES	NK
15	YES	YES	NK	YES	NK	YES	NK	NO	YES	NK
16	YES	YES	NK	YES	NK	YES	NK	YES	YES	NK
17	YES	YES	NK	YES	NK	YES	NK	YES	YES	NK
18	YES	YES	NK	NK	NK	NK	NK	NO	NK	NK
19	YES	YES	NK	NK	YES	NK	YES	NO	NK	NK
20	YES	YES	NK	YES	NK	YES	NK	YES	YES	NK
21	YES	YES	NK	NK	NK	NK	NK	NO	YES	NK
22	YES	YES	NK	NK	YES	NK	YES	NO	NK	NK
23	YES	YES	NK	YES	NK	YES	NK	YES	NK	NK
24	YES	YES	NK	YES	YES	YES	YES	YES	NK	NK
25	YES	YES	NK	NK	NK	NK	NK	YES	NK	NK
26	YES	YES	NK	YES	NK	YES	NK	YES	YES	NK

27	YES	YES	NK	YES	NK	YES	NK	YES	YES	NK
28	YES	YES	NK	YES	NK	YES	NK	YES	YES	NK
29	YES	YES	NK	YES	YES	YES	YES	YES	YES	NK
30	YES	YES	NK	YES	NK	YES	NK	YES	YES	NK
31	YES	YES	NK	YES	YES	YES	YES	YES	YES	NK
32	YES	YES	NK	NK	NK	NK	NK	NO	NK	NK
33	YES	YES	NK	NK	NK	NK	NK	NO	NK	NK
34	YES	YES	NK	YES	YES	YES	YES	YES	YES	NK
35	YES	YES	NK	YES	YES	YES	YES	NO	YES	NK
36	YES	YES	NK	YES	NK	YES	NK	YES	YES	NK
37	YES	YES	NK	NK	NK	NK	NK	NK	YES	NK
38	YES	YES	NK	YES	YES	YES	YES	YES	YES	NK
Percentage of Yes	100%	97,37 %	18,42 %	65,79 %	42,11%	65,79 %	42,11 %	63,16%	73,68%	0%

YES=YES
NO=NO
NK=NOT KNOWN
